# Keratin 17-positive Civatte bodies in oral lichen planus—distribution variety, diagnostic significance and histopathogenesis

**DOI:** 10.1038/s41598-020-71496-8

**Published:** 2020-09-03

**Authors:** Tatsuya Abé, Norio Kitagawa, Shohei Yoshimoto, Satoshi Maruyama, Manabu Yamazaki, Tetsuichiro Inai, Shuichi Hashimoto, Takashi Saku

**Affiliations:** 1 Division of Molecular and Diagnostic Pathology, Niigata University Graduate School of Medical and Dental Sciences, Niigata, Japan; 2 Oral Pathology Section, Department of Surgical Pathology, Niigata University Hospital, Niigata, Japan; 3 Division of Oral Pathology, Department of Tissue Regeneration and Reconstruction, Niigata University Graduate School of Medical and Dental Sciences, Niigata, Japan; 4 Department of Morphological Biology, Division of Biomedical Sciences, Fukuoka Dental College, 2-15-1 Tamura, Sawara-ku, 951-8514 Japan

**Keywords:** Oral anatomy, Inflammation, Diseases

## Abstract

Although emergence of keratin 17 (K17) and reciprocal loss of K13 are immunohistochemical hallmarks for oral mucosal malignancy, we report here findings of K17-positive (+) speckles, possibly equivalent to Civatte bodies, in benign oral lichen planus. Sixty-two biopsy samples from oral lichen planus cases were subjected to immunohistochemical examinations to analyze the distribution as well as histopathogenesis of Civatte bodies. K17 was irregularly positive among oral lichen planus-affected epithelial cells, and K17-positive (+) filamentous structures were irregularly distributed within the cytoplasm in confocal images. K17+ speckles were identified as Civatte bodies, and they were mainly distributed in the interface between epithelial cells and lymphocytic infiltrates (type A, 52.8%), followed by distribution within the epithelial layer (type B, 24.7%) or within the lamina propria with lymphocytic infiltration (type C, 22.5%). Apoptotic figures were often engulfed by macrophages and clearly distinguished from Civatte bodies by the presence TUNEL signals. These results indicate that K17 is a sensitive immunohistochemical marker for Civatte bodies and useful for differential diagnosis of oral lichen planus from other oral mucosal lesions. Civatte bodies are generated from denucleation of K17+ epithelial cells during the process of cell death via dyskeratosis, which is possibly related to blood capillary collapse.

## Introduction

In previous studies, we have emphasized that reciprocal immunohistochemical profiles between keratin (K) 13 (loss) and K17 (emergence) are valuable for histopathological differential diagnosis of oral carcinoma in-situ (CIS) from epithelial dysplasia, which is challenging when only using hematoxylin and eosin (HE) stained sections^1,2^. Emergence of K17 has been shown to be required for cell proliferation of cancer cells and their larger size^3^. Additionally, our recent proteome analysis findings confirmed that loss of K13 and emergence of K17 were more exclusively specific to oral CIS and squamous cell carcinoma (SCC) as compared to other keratin subtypes^4^. We have also noticed the presence of K17-positive (+) cell-debris-like speckles scattered in tissue specimens obtained from oral lichen planus cases, which were obviously benign and not candidates for differential diagnosis from any malignant lesions.

Civatte bodies, also known as colloid bodies or hyaline bodies, are regarded as a diagnostic hallmark for lichen planus^5^. Historically, they were first clearly described and illustrated as “corps hyalins” by Achille Civatte in the lichen planus section of his dermatopathology atlas published in 1957^6^, though the presence of Civatte bodies themselves in lichen planus was first described by Sabouraud in 1912^7^, according to the review by Burgdorf and Plewig^8^. Following presentation of Civatte’s atlas^6^, the presence of colloid/hyaline bodies has come to be recognized as one of the five classic and important histopathological findings of oral lichen planus, in addition to (1) hyperkeratosis or parakeratosis, (2) saw-toothed rete pegs, (3) superficial infiltrate of lymphocytes, and (4) basal cell liquefaction^9^. Hyperkeratosis, especially hyperorthokeratosis with hypergranulosis, are typical of skin lesions, but not always features of oral lichen planus^10,11^. The term “Civatte body” itself as replacement for colloid/hyaline body seems to have become widely accepted in the late 1960s, as seen in Montgomery’s textbook of dermatology, though that eponym is not specifically referred to in the text^12^.

Ebner and Gebhart had previously disclosed in their ultrastructural study conducted in 1972 that Civatte bodies are of epithelial origin, based on evidence showing that 8- to 10-nm sized filaments and cell organelles, but no nuclear components, were packed inside them^13^. However, Jean Civatte presented the initial immunohistochemical finding of immunoglobulin (Ig) M localized in Civatte bodies^14^. Thereafter, Ig-absorbed Civatte bodies were shown to be epithelial cell debris based on immunohistochemistry findings of keratin subtypes in skin lesions, including lichen planus^15–17^. Contrary to skin lesions, there has only been a small number of immunohistochemical investigations of keratin (wide spectra) presented and no keratin subtypes have been clarified for Civatte bodies of oral lichen planus^18–20^, though the immunohistochemical profiles of oral mucosal epithelial cells are known to be different from those of skin epidermal cells.

Based on our findings noted above showing that K17+ speckles in oral lichen planus were identical to Civatte bodies, we speculated that K17 immunohistochemistry would be useful to demonstrate Civatte bodies and might also be effective for histopathological differential diagnosis of oral lichen planus. In the present study, we investigated tissue samples obtained from oral lichen planus patients to determine the exact distribution profile of Civatte bodies and elucidate the background of K17 emergence in oral lichen planus-affected epithelial cells. From those results, we discuss the possible histopathogenesis of Civatte bodies as well as their mode of cell death, which is different from apoptosis.

## Materials and methods

### Clinical samples

Biopsy specimens from a total of 62 cases of oral lichen planus were collected for the present study from the surgical pathology files of the Division of Oral Pathology, Niigata University Graduate School of Medical and Dental Sciences, that had been obtained during the 12-year period from 2008 to 2019, as well as from the files of Fukuoka Dental College Hospital from 2018 to 2019, following a histopathological review. As controls, 10 surgical excision samples of CIS of the oral mucosa containing normal mucosal components were subjected to immunohistochemical examination. The specimens were fixed in 10% formalin and routinely embedded in paraffin. Serial sections were cut into 4-µm slices from paraffin blocks and subjected to hematoxylin and eosin (HE) staining and immunohistochemistry. The present experimental protocol for analyzing surgical materials was reviewed and approved by the ethics review boards of Niigata University Graduate School of Medical and Dental Sciences (approval number 2019-0327) and Fukuoka Dental College (approval #487). All experiments were performed in accordance with the relevant guidelines and regulations. The need for written informed consent was waived by the institutional ethics committees with informed consent obtained in the form of an opt-out form available on the web-site of each hospital.

### Immunohistochemistry

Immunohistochemistry was performed using the ChemMate Envision system (Dako, Glostrup, Denmark), as described elsewhere^1–3^. For K10, K13, K17, and K19, sections were pretreated as previously described^2,21^. For cleaved caspase 3 (caspase 3) and CD68, sections were autoclaved at 121 °C for 10 min in Tris–EDTA buffer (pH 9.0) and citric acid buffer (pH 6.0), respectively. For CD31, sections were pre-treated with 0.2% trypsin (type II, Sigma Chemical Co, St. Louis, MO, USA) in 0.01 M Tris–HCl (pH 7.6) containing 0.1% CaCl_2_ for 30 min at 37 °C. For the control experiments, the primary antibodies were replaced with pre-immune IgGs. Terminal deoxynucleotidyl transferase dUTP nick end labeling (TUNEL) was performed as described elsewhere^4^. K17+ speckles were compared with Civatte bodies seen in serial HE-stained sections.

### Immunofluorescence

For double-immunofluorescence examinations of caspase 3 and CD68, Alexa Fluor 568-conjugated goat anti-rabbit IgG antibody (Invitrogen, Thermo Fisher Scientific, Waltham, MA, USA) and Alexa Fluor 488-conjugated goat anti-mouse IgG antibody (Invitrogen), respectively, were used as the secondary antibodies. For caspase 3 and hemoglobin, the anti-caspase 3 was pre-labeled with Alexa Fluor 568 (ZenonTM kit, Thermo Fischer Scientific), while Alexa Fluor 488-conjugated goat anti-rabbit IgG was used as the secondary antibody for hemoglobin. Labeled sections were covered with Immunoselect Antifading Mounting Medium DAPI (Dianova, Hamburg, Germany). K17 immunofluorescence was performed for confocal microscopy to investigate K17+ keratin filament distribution within oral squamous epithelial cells on tissue sections by an indirect method using Alexa Fluor 568-conjugated goat anti-mouse IgG (H + L) (Invitrogen) as the secondary antibody, as described elsewhere^22^. The slides were examined with an LSM710 confocal laser scanning microscope (Zeiss, Oberkochen, Germany). Z-stack images were obtained at constant Z intervals of 0.5–1.0 µm. Images and montages were generated using Zeiss Zen software (Zeiss) and Adobe Photoshop (Adobe Systems, San Jose, CA, USA).

### Statistical analysis

A chi-squared test was used to determine whether there was a difference for Civatte body distribution among the three tissue zones using SPSS 22 (IBM Corp., Armonk, NY, USA). A *P*-value < 0.01 was considered to indicate significance.

## Results

### Keratin immunohistochemistry for oral epithelial conditions

**Figure 1:**
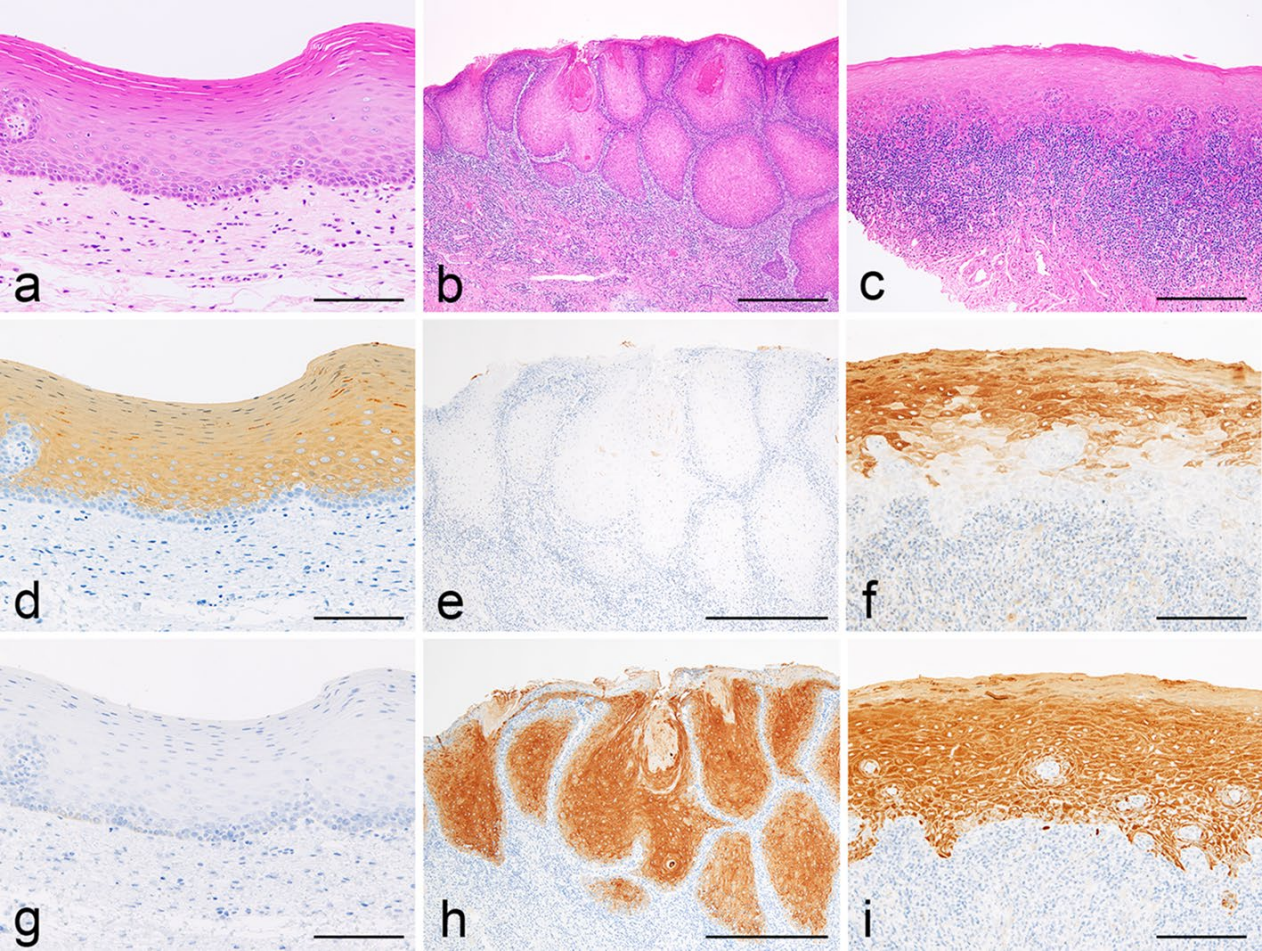
Comparative immunohistochemical profiles of keratin subtypes 13 and 17 in three different oral mucosal conditions. (**a,d,g**) Normal epithelium; (**b,e,f**) carcinoma in-situ (CIS); (**c,f,i**) oral lichen planus. Hematoxylin and eosin (HE) (**a-c**) and immunoperoxidase stains for keratin (K) 13 (**d–f**) and K17 (**g–i**), with hematoxylin counterstaining. (**a**,**d**,**g**) × 200; (**b**,**e**,**f**) × 100; (**c**,**f**,**i**) × 100. Scale bars, (**a**,**d**,**e**,**f**,**g**,**h**,**i**) 200 µm; (**b**,**c**) 50 µm. Normal epithelium (**a**) was positive for K13 (**d**) but not for K17 (**g**). In CIS (**b**) and squamous cell carcinoma (not shown), K13 had completely disappeared (**e**), with K17 instead strongly emerged with faint keratin pearls (**h**). In oral lichen planus (**c**), K13 was not seen in the lower half of the epithelium but was irregularly positive in the upper half (**f**). K17 was also positive in the epithelial layer with lower levels of intensity in the basal zone. Small K17-positive (+) dot-like speckles were scattered along the epithelial interface (**i**).

As a control, we performed immunohistochemistry to compare K13 and K17 under two different conditions of the oral mucosa; normal epithelia (Fig. 1a) and CIS (Fig. 1b), in addition to oral lichen planus (Fig. 1c). As previously documented, most epithelial cells, except for the basal and parabasal cells, namely those in the third and upper layers, of the normal mucosa (Fig. 1a) were positive for K13 (Fig. 1d), whereas none were positive for K17 (Fig. 1g). In contrast, in CIS (Fig. 1b), K13 had disappeared from neoplastic cells (Fig. 1e), and K17 reciprocally emerged in them, while keratinized cells were only weakly positive (Fig. 1h). In oral lichen planus (Fig. 1c), K13 was not found in the lower half of the epithelium, though was irregularly positive in the upper half (Fig. 1f), whereas K17 was positive in the whole epithelial layer with lower intensity in the basal zone. In addition, small speckles with K17+ staining were scattered along the epithelial interface (Fig. 1i), which we considered to be identical to Civatte bodies.

In addition to K13 and K17, we also performed immunohistochemistry for K19, and K10, known to be specific for basal cells, and dysplastic orthokeratotic cells, respectively, of the oral mucosa^1,2,21,23^. Neither of those keratin subtypes were found localized in Civatte bodies. Furthermore, lichen planus-affected epithelial cells were not positive for K19 and K10 (data not shown).

### Keratin immunohistochemistry for Civatte bodies

**Figure 2:**
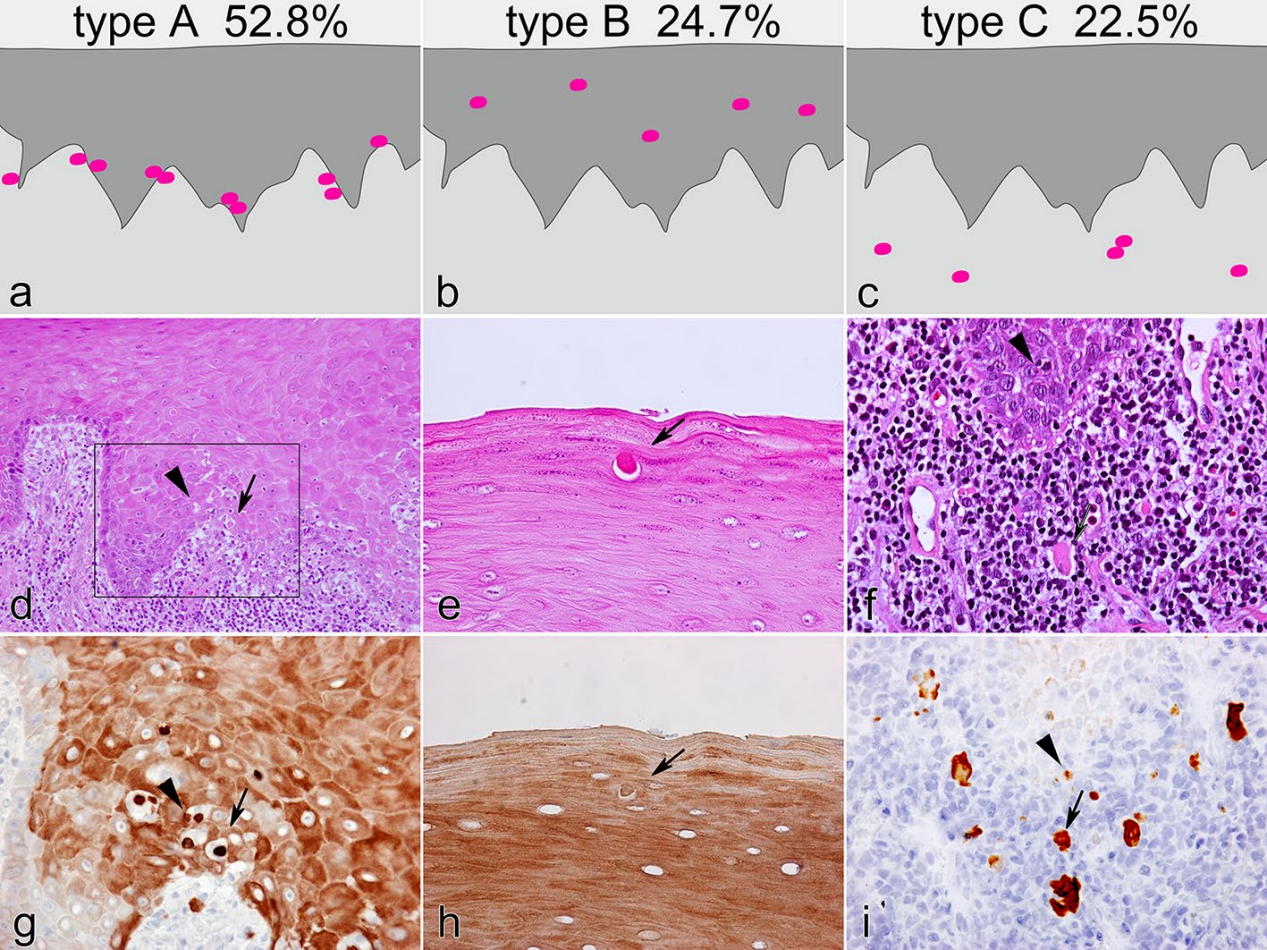
Three modes of Civatte body distribution revealed by immunohistochemistry for K17 in oral lichen planus. (**a**) Type A, at interface between epithelial layer and underlying lymphoid cell infiltrates. (**b**) Type B, in intraepithelial layer. (**c**) Type C, in lamina propria with band-like infiltration of lymphoid cells. HE (**d**-**f**) and immunoperoxidase stains for K17 (**g**–**i**), with hematoxylin counterstaining. (**d**) × 100; (**e**–**i**) × 400; arrows, Civatte bodies; arrowheads, apoptotic bodies; (**g**) square in (**d**) showing a serial section; scale bars, (**d**) 200 µm; (**e**–**g**) 50 µm. Not all Civatte bodies were recognized in HE-stained sections (**d**–**f**), whereas they were easily visualized by immunohistochemistry for K17, including number, shape, and location (**g**–**i**). Civatte bodies were round to oval in shape, and more intensely stained and clearly isolated from surrounding epithelial cells, which were also irregularly positive for K17 (type A, **d**,**g**). In contrast to Civatte bodies, apoptotic bodies were clearly distinguished because of fine and punctuated K17 signals (**d**,**g**). Civatte bodies were also found within the upper prickle to keratinized layer (type B, **e**,**h**) and in the lamina propria with lymphoid cell infiltration (type C, **f**,**i**). Type C Civatte bodies were occasionally fused to form larger and irregular shapes. Apoptotic bodies were only observed within the epithelial layer, especially along the basal zone (**i**). In 56 of the 62 examined cases of lichen planus, type A was most frequently seen (52.8%), followed by type B (24.7%) and type C (22.5%).

Immunohistochemical results revealed round or ovoid-shaped eosinophilic cell debris without nuclei, Civatte bodies, that were strongly positive for K17. Among all cases (62 biopsy specimens) of oral lichen planus investigated, 56 (90.3%) contained Civatte bodies. Furthermore, K17-immunohistochemistry enabled visualization of Civatte bodies in three different locations; the interface between the epithelial layer and underlying lymphoid cell infiltrates (type A, Fig. 2a), the intraepithelial layer (type B, Fig. 2b), and the lamina propria with a band-like infiltration of lymphoid cells (type C, Fig. 2c). Type A bodies were most frequently observed and relatively easily recognized even in HE-stained sections (Fig. 2d, arrow), and they were revealed as deeply stained and punctuated forms in immunohistochemical findings (Fig. 2g, arrow). Apoptotic figures were frequently noted in the vicinity of type A Civatte bodies in the epithelial basal zone (Fig. 2d, arrowhead), though the fine dot-like K17+ signals of the apoptotic bodies were distinct from the larger round/ovoid-shaped Civatte bodies (Fig. 2g, arrowhead). In addition to Civatte bodies, epithelial cells were irregularly positive for K17, with uneven staining intensity among them and variances between areas, as well as irregularities within individual cells (Fig. 2g,i). Type B bodies were observed as isolated round- or ovoid-shaped speckles within the prickle cell layer or keratinized layer (Fig. 2e), and their immunohistochemical staining intensity was not always as conspicuous as surrounding K17+ epithelial cells (Fig. 2h). Type C bodies were found in the background of lymphoid cell infiltration and not easily recognized in HE-stained sections (Fig. 2f, arrow), whereas they were clearly shown in K17 immunohistochemical findings (Fig. 2i, arrow). Apoptotic bodies were located within the epithelial layer or along the basal area, but not in the lamina propria (Fig. 2f,i, arrowheads). The number of evaluatable K17+ Civatte bodies from the 56 oral lichen planus cases (biopsy specimens) containing Civatte bodies totalled 481, while frequency based on location was 254 (52.8%) in type A (basal zone); 119 (24.7%) in type B (intraepithelial), and 108 (22.5%) in type C (lamina propria) (Fig. 2, upper row). There was a statistically significant predominance for Civatte body location in the basal zone (type A) (*P* < 0.01). Civatte bodies within the epithelial layer (type A and B) were round/ovoid shaped, while type C bodies did not always have a smooth outline, but rather showed a rough margin, irregular shape, and size variations. We did not analyze the relationship between distribution modes of Civatte bodies (type A, B, and C) and clinical characteristics of the patients.

### Investigation for apoptosis in Civatte bodies

**Figure 3:**
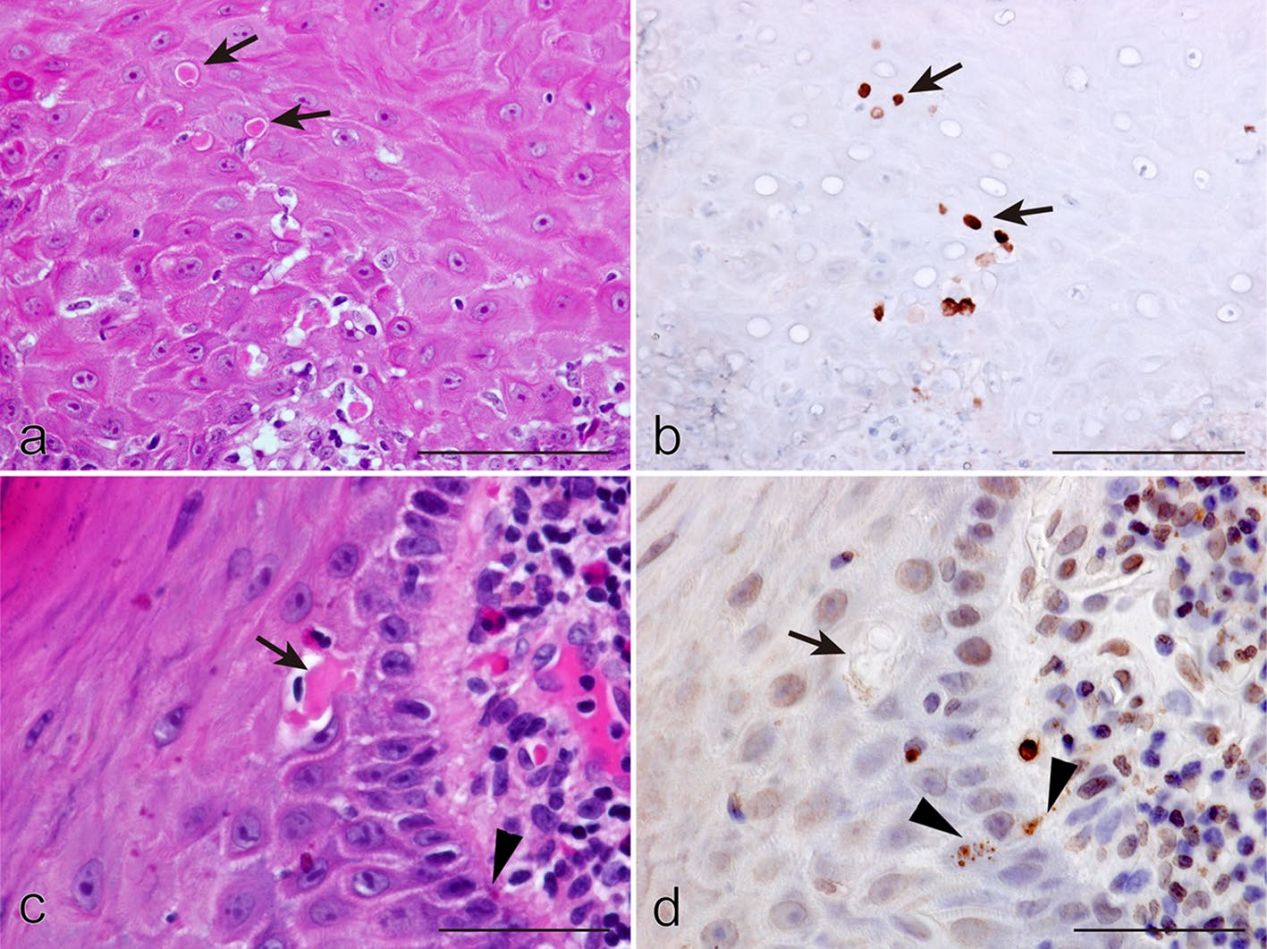
Immunohistochemical profiles of cleaved caspase 3 (caspase 3) and TdT-mediated dUTP nick end labeling (TUNEL) signals in oral lichen planus. HE (**a,c**) and immunoperoxidase stains for caspase 3 (**b**) and TUNEL (**d**), hematoxylin counterstain. Arrows, Civatte bodies; arrowheads, apoptotic bodies. (**a**,**b**) × 400; (**c**,**d**) × 710; scale bars, (**a**,**b**) 50 µm, (**c**,**d**) 25 µm. Type A and B Civatte bodies (**a**, arrows) were diffusely positive for caspase 3 (**b**, arrows). Staining intensity varied, different from the even and intense staining for K17 (Fig. 1g–i), indicating that caspase 3 gradually disappeared from Civatte bodies. In contrast, Civatte bodies (**c**, arrow) were not labeled for TUNEL (**d**, arrow), though TUNEL+ signals were recognized in scattered apoptotic bodies, which also had granular staining for caspase 3 (**b**), in the vicinity (**d**, arrowheads).

**Figure 4:**
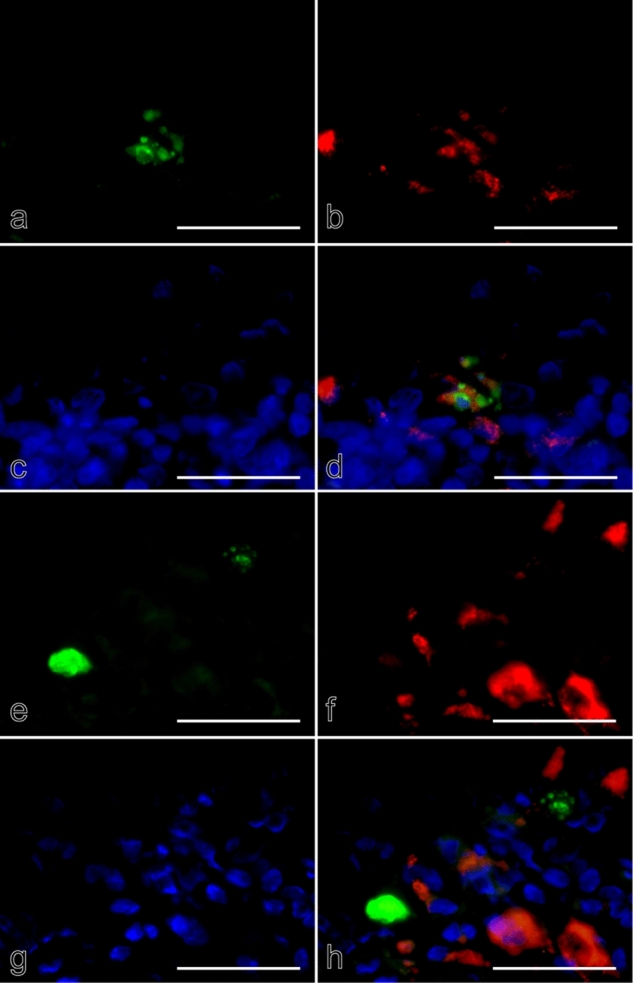
Comparative immunohistochemical profiles of caspase 3 and CD68 in oral lichen planus. (**a–d**) Apoptotic cells; (**e–h**) Civatte bodies. Double immunofluorescence for caspase 3 (**a,e**; green) and CD68 (**b,f**; red), counterstained with 4′,6-diamidino-2-phenylindole (DAPI) (**c,d,g,h**; blue). (**a**–**d**) × 1,000; scale bar, 50 µm. Apoptotic cells in the form of apoptotic bodies were recognized based on fine to coarse dot-like signals for caspase 3 (**a**,**e**, green), and did not show obvious DAPI-binding except for larger dot forms (**c**,**e**; blue, a small number of dots). The caspase-3+ signals were overlapped with staining for CD68, indicating that apoptotic bodies were engulfed by macrophages (**b**, red). Apoptotic body (**a**,**c**) phagocytosis by macrophages (**b**) was clearly demonstrated when the three immunofluorescence images were merged (**d**). In contrast, Civatte bodies were densely labeled for caspase 3 with a mottled appearance (**e**), different from the dot-like staining pattern of apoptotic bodies. CD68+ macrophages were not recruited towards Civatte bodies (**f**). The differences between Civatte bodies and apoptotic bodies were sharply demonstrated in merged images (**h**).

Based on findings from our K17 analysis showing Civatte bodies to be epithelial cell debris, we next attempted to confirm if they were in an apoptotic process. Immunohistochemical results showed that Civatte bodies were diffusely positive for caspase 3 with different levels of staining intensity among them (Fig. 3a,b). No TUNEL signals were noted in Civatte bodies, whereas apoptotic bodies (Fig. 3c,d; arrowheads) seen in the vicinity of Civatte bodies (Fig. 3c,d; arrow) showed TUNEL+ signals. Apoptotic bodies were also positive for caspase 3, though the staining was not homogeneous but rather a fine granular-to-coarse dotted intensity (Fig. 4a,d; green), which were different from the staining of Civatte bodies (Fig. 4e, h; green). Additionally, apoptotic bodies, especially fine forms, were not always positive for DAPI (Fig. 4c) and engulfed by CD68+ macrophages (Fig. 4b,d; red). In contrast, caspase 3 + Civatte bodies without nuclear components (Fig. 4e,g; green) were not phagocytosed by macrophages (Fig. 4f,h; red). Based on these findings, we concluded that Civatte bodies are different from apoptotic cells.

### Relationship between blood capillary vessels and Civatte bodies

**Figure 5:**
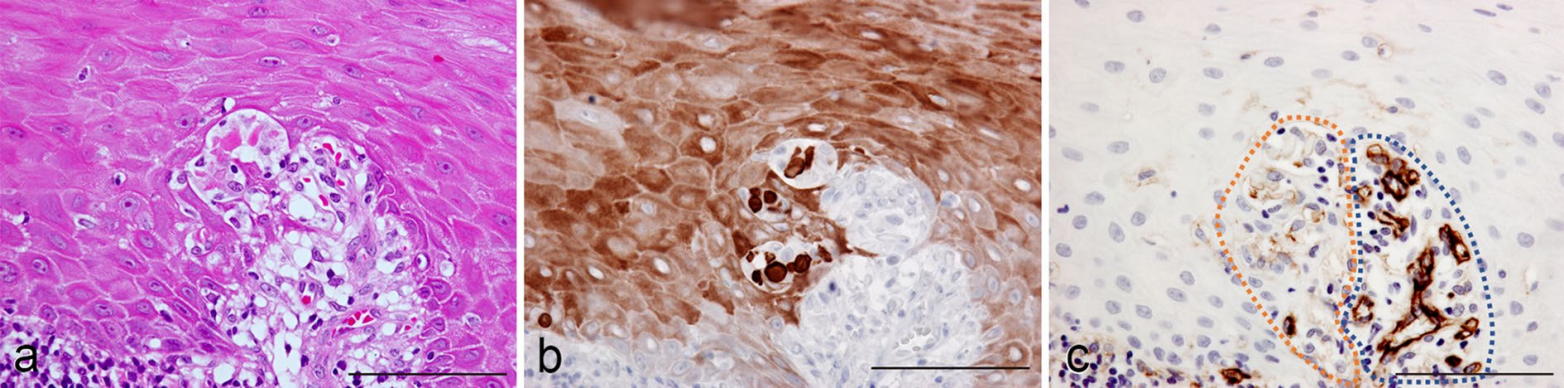
Blood capillary collapse related to Civatte body generation in oral lichen planus. HE (**a**) and immunoperoxidase stains for K17 (**b**) and CD31 (**c**), hematoxylin counterstain. (**a–c**) × 400; scale bars, 50 µm. When Civatte bodies (**a**), which were positive for K17 (**b**), were observed in the epithelial interface zone, capillary loop structures around them were obscure because epithelial cells were detached from each other and distorted in shape in the presence of lymphoid cells (**a**). Around the Civatte bodies, blood capillary loops were not clearly demonstrated by immunohistochemistry for CD31 (circled with red dotted line), while blood capillaries were definitely revealed by CD31 in areas without Civatte bodies, where basal cell alignment was preserved (blue dotted line) (**c**).

**Figure 6:**
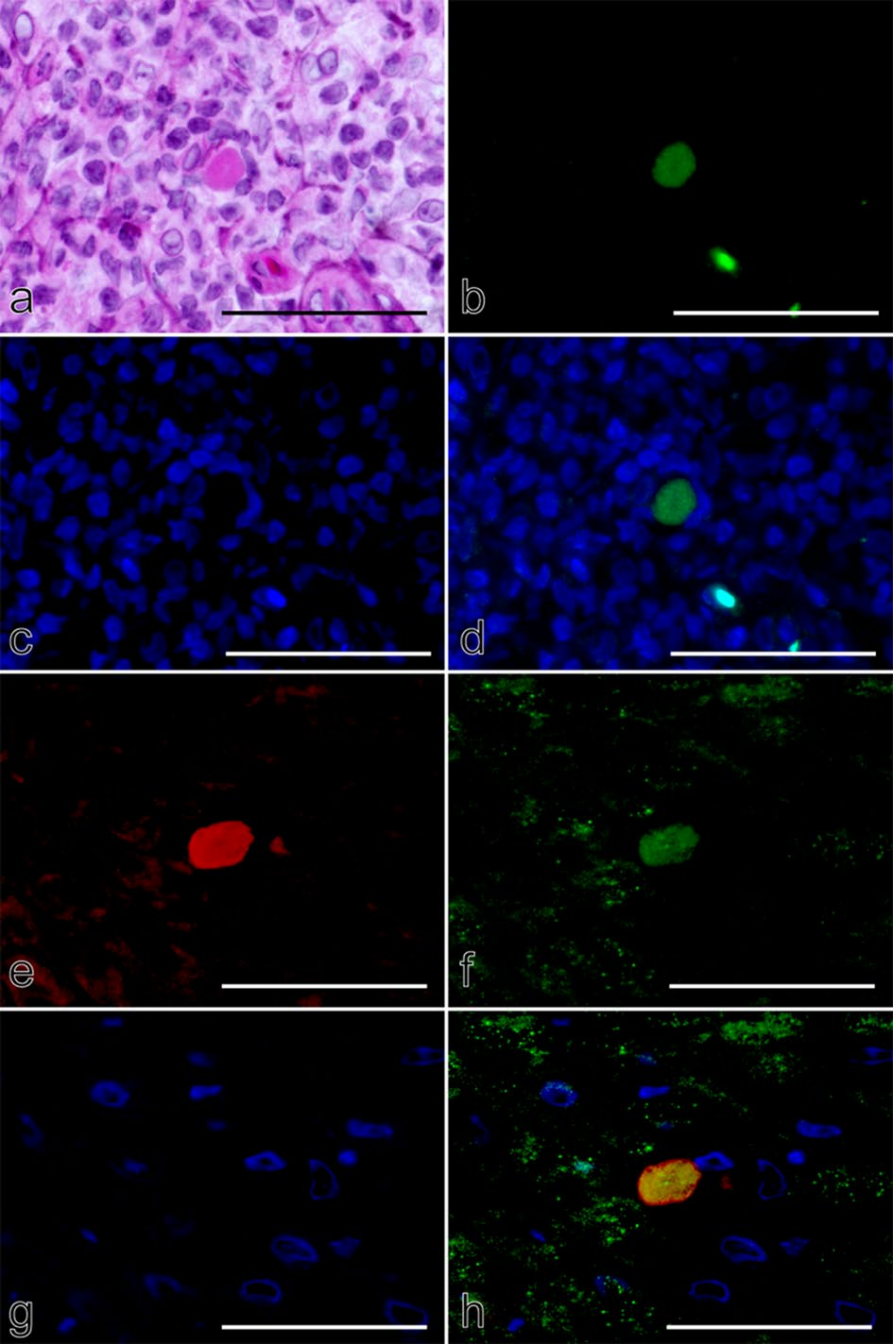
Immunohistochemical profiles for hemoglobin in Civatte bodies of oral lichen planus. (**a**) HE and double immunofluorescence stains for hemoglobin (**b,d,f,h**; green), DAPI (**c,d,g,h**; blue), and caspase 3 (**e,h**; red). (**a–h**) × 1,150; scale bar, 50 µm. Type C Civatte body within lymphocytic infiltrates (**a**) was densely positive for hemoglobin with a mottled appearance (**b**, green) but did not show DAPI binding (**c**,**g**; blue). As compared to the brilliant hemoglobin signals from erythrocytes (**b**, right lower zone), the staining intensity in Civatte bodies was rather low (**b**,**d**). Caspase 3 was also intensely labeled in Civatte bodies (**e**, red), which were simultaneously positive for hemoglobin (**f**, green). Fine dot-like signals for hemoglobin indicated hemolytic fragments from erythrocyte extravasation over the epithelial layer (**f**, green), while hemoglobin staining intensity of erythrocytic fragments was stronger than that of Civatte bodies (**f**,**h**).

Since extravasated erythrocytes were frequently seen around type A Civatte bodies, with blood vessels not clearly recognized in HE-stained sections (Fig. 5a,b), we attempted to determine the relationship between them and blood capillary vessels. Immunohistochemistry for CD31 clearly revealed intact blood vessel walls of capillary loops in the lamina propria (Fig. 5c, blue circle), whereas blood vascular endothelial cells in areas around Civatte bodies were scarcely or very faintly apparent (Fig. 5c, red circle), possibly indicating that those blood vessels were collapsed. To demonstrate the presence of erythrocytes, we performed immunohistochemistry for hemoglobin. In addition to erythrocytes, Civatte bodies were also strongly positive for hemoglobin (Fig. 6a–d,f), which was confirmed by simultaneous positivity for caspase 3 (Fig. 6e,h). Prickle cells surrounding Civatte bodies occasionally contained hemoglobin+ granules in cytoplasm, which were considered to be hemophagocytosis figures, as reported in cases of oral malignancy^23^ (Fig. 6f,h). These results indicated that Civatte bodies are generated via a type of keratinization due to oxidation through capillary loop collapse, and then become absorbed and retain hemoglobin primarily derived from hemolysis during the process of formation.

### Confocal microscopic analysis of K17 immunolocalization profiles

**Figure 7:**
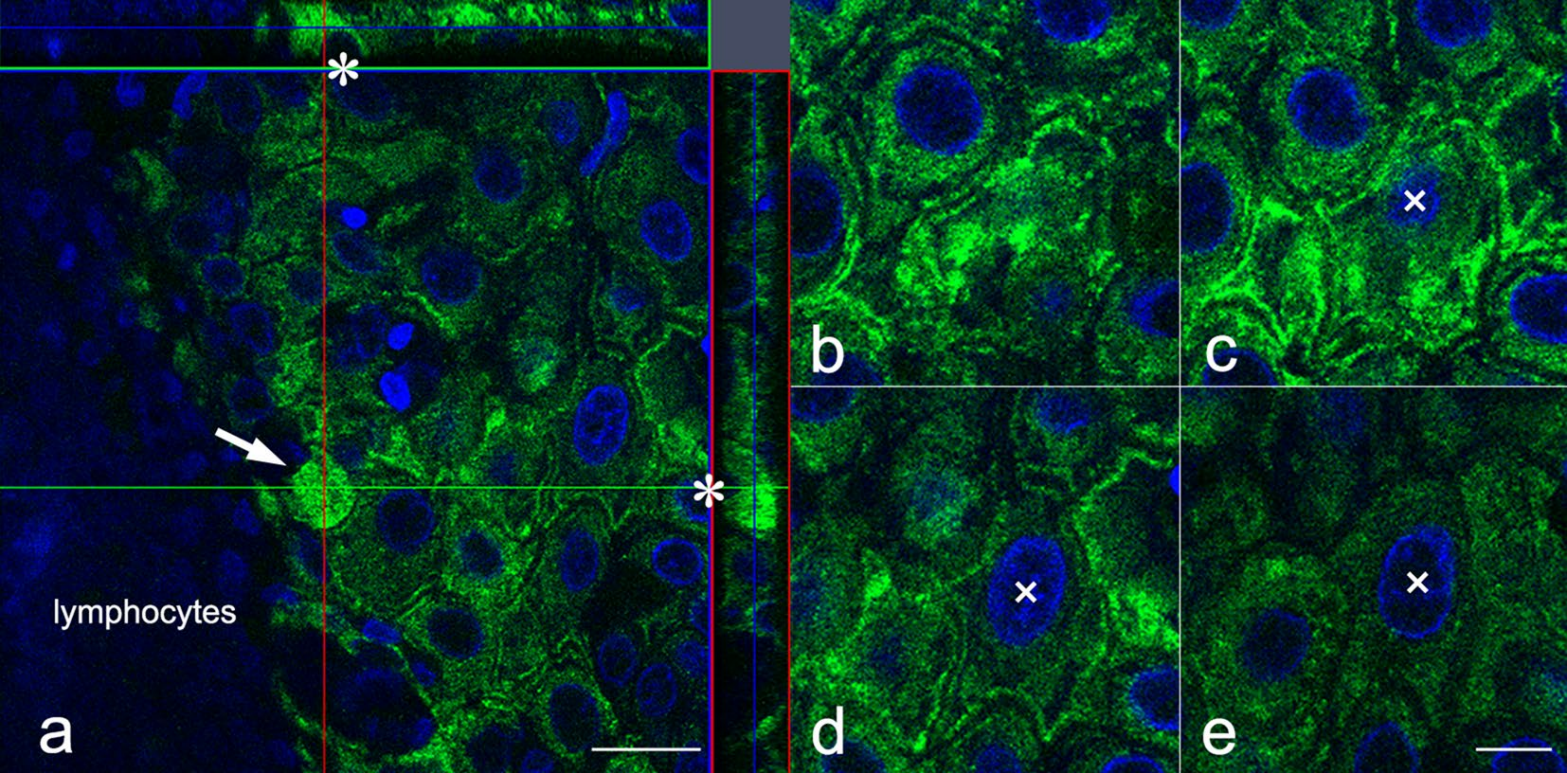
Confocal microscopy findings of K17 in oral lichen planus. (**a**) Initial stage of Civatte body formation with Z-axis stacks (upper window, green line section; right window, red line section). (**b–e**) Irregular arrangement of K17+ cytoskeletal filaments in different focal planes (×, same cell nucleus). (**a**) × 620; scale bar, 20 µm, (**b–e**) × 1030; scale bar, 10 µm. Shown is a representative singular round-shaped epithelial cell packed with condensed K17+ signals visualized in the interface of the epithelial layer and lymphoid cell infiltrate (indicated as “lymphocytes”), and considered to be an immature Civatte body (a, arrow). When the Z-axis stacks were observed, there was a round shaped empty space that seemed to have been its nucleus because of no DAPI binding (**a**, indicated by asterisks^*^). In the vicinity of this Civatte body, epithelial cells were positive for K17 with different staining intensities (**a**,**b**–**e**). In each Z-axis slice, K17+ filaments were condensed along the cell membrane (**c**–**e**; ×, same cell nucleus), whereas they were irregularly condensed or had a mottled appearance in areas between the cell membrane and nuclei. Those filamentous conglomerates were adhered to the cell membrane, and occasionally formed an irregularly shaped net- or ring-like structure (**b**–**e**).

The uneven and varied K17 staining intensity among epithelial cells of lichen planus foci (Fig. 2g) was confirmed by confocal microscopy. Figure 7a shows a representative type A Civatte body in the interface zone between the epithelial layer and subepithelial lymphocytic infiltrate (Fig. 7a, indicated as “lymphocytes”). As seen in the Z-axis stacks in the upper (green line slice) and right (red line slice) squares of that panel, K17+ cytoskeletal filaments were condensed above a nuclear space that lacked DAPI binding (Fig. 7a, indicated by a long arrow and asterisks) and there was no cellular rim around the thick filamentous conglomerates. These findings indicated an initial stage of Civatte body formation and that Civatte bodies are not the same as apoptotic products, but rather denucleated cell debris, in which K17+ filaments are condensed, as also seen with keratinization. Many of the prickle cells were outlined by K17+ thick cell borders, while the width of the K17+ filaments was not even, indicating irregular fusion of cytoskeletal filaments to the membrane. In addition, K17+ filaments were irregularly condensed within the cytoplasm, the same as seen in the different focal planes, e.g., around the nuclei in some cells, or between the membrane and nuclei in others (Fig. 7b–e). Furthermore, they occasionally formed irregular circular structures (Fig. 7c). These results were considered to indicate abnormal keratinization (dyskeratotic) products.

## Discussion

The present results are the first to show that Civatte bodies can be clearly demonstrated by K17 immunohistochemistry. Furthermore, those bodies were found to be disseminated not only in the epithelial interface with the lamina propria but also in subepithelial lymphoid cell infiltrates, as well as within prickle-cell and keratinized-cell layers. In addition, Civatte bodies were apparently different from apoptotic figures because they do not exhibit TUNEL signals, but are rather formed by a type of single cell keratinization process with denucleation of K17+ squamous epithelial cells, in which K17  cytoskeletal filaments are irregularly arranged. Civatte bodies lacked cell membranes and had an ability to absorb hemoglobin, indicating their exposure to hemorrhage. The switching from K13 to K17 in oral lichen planus-affected epithelial cells suggests that those were either activated or damaged by lymphocytic infiltration, though they were not involved in a neoplastic process because they lacked other immunohistochemical profile factors indicating malignancy.

As noted previously, Civatte bodies were not initially recognized as epithelial cell debris in their historical context but rather regarded as degenerated forms of histiocytes, plasma cells, or epithelial cells, as described in Lever’s textbook, 4th edition (1967)^24^, which also referred to articles presented by Gougerot and Civatte^25^, and Golttz and Hult^26^. The change in understanding occurred because Eber and Gebhart reported electron microscopy findings showing that Civatte bodies in lichen planus of the skin had an epithelial origin^13^. Similar electron microscopic observations were then later demonstrated for Civatte bodies in oral lichen planus cases^27–30^.

Following the first report of IgM+ Civatte bodies by Jean Civatte^14^, Biermann and Rauterberg reported that Ig-coated Civatte bodies of skin lesions were simultaneously immunopositive for K10/11, K8/18, and K13, with the latter two termed fetal keratins because of their transient expressions in fetal epidermis and results showing that skin Civatte bodies were also immunolabeled with the basal cell-specific monoclonal antibody BL7^16^. Gürbüz et al. showed altered keratin expression profiles including mild K17 immunoreactivity in keratinocytes in lichen planus of the skin, though they did not investigate Civatte bodies^15^. Prior to this article, no detailed immunohistochemical investigation of keratin subtypes in Civatte bodies in oral lichen planus has been reported, though the immunohistochemical profiles for keratin subtypes of oral mucosal epithelial cells are different from those of the epidermis^31^. Pihlman et al. only demonstrated simultaneous immunolocalization of fibrin, IgM, and keratin (wide spectra) within Civatte bodies^19^.

In our previous studies, we confirmed reciprocal switching from K13 to K17 and the emergence of K10 in oral malignancy^1,2,20^. The present results showed that the former phenomenon also occurs in oral lichen planus, though switching was incomplete, as shown in Fig. 1. In contrast to skin lichen planus, neither K13 nor K19 was found to be positive in Civatte bodies in the present study. These results indicate that Civatte bodies are not derived from normal prickle cells (K13+) nor normal basal cells (K19+), but rather from epithelial cells that already contain K17+ cytoskeletal filaments. Interestingly, aberrant keratin subtypes such as fetal K13 and K8/18, which were not found to be expressed in normal epidermis, were shown to be immunolocalized in skin Civatte bodies, in addition to K10, a marker of normal epidermal differentiation^15^. Therefore, lack of K10 expression in oral lichen planus-affected epithelial cells as well as Civatte bodies demonstrated in the present study indicates that epithelial cell resources for Civatte bodies are not related to orthokeratotic dysplasia cells or dyskeratotic cancer cells, both of which are positive for K10 but not for K13 or K19^21^.

It should be emphasized that K13 is regarded as fetal keratin and K10 as postnatal keratin in skin^16^, though K13 is expressed in normal oral mucosa. Fetal epidermal K13 is now known to be expressed in the epidermis of patients with psoriasis, in which autoantibodies against K13 are also present^32^. In addition to K13, ectopic K17 is also expressed in the epidermis of individuals with psoriasis, in which epidermal cells are considered to be damaged and activated by external stimuli such as infection, oxidative stress, and drugs. Damaged or activated keratinocytes are monitored by dendritic cells, which mediate recruitment of Th1 or Th17 lymphocytes. Secretion of TNF-α, INF-γ, IL-17, or IL-22 from those T cells induces epidermal keratinocytes to express K17 via STAT1/STAT3 pathways^33^. Thus, some similar cell machineries may mediate the incomplete reciprocal switching to K17 from K13 in oral lichen planus, though no direct evidence for that has yet been shown^11,34^. In oral malignancies, the collaboration between K17 and 14-3-3 sigma modulates proliferation of oral SCC cells^3^, while it remains unknown whether this is also true for oral lichen planus.

As for the alternative change of keratin subtypes occurring in damaged or activated epithelial cells, our confocal microscopic study is the first to demonstrate that aberrant K17+ filaments characteristically show an irregular arrangement in oral lichen planus-affected epithelial cells. We previously reported that K17+ filaments were found to be distributed in a limited area in ring- or net-like shapes in the HaCaT cell line, which are immortalized keratinocytes, following exposure to nisin, a product of *Lactococcus lactis subsp. lactis*^22^, one of the resident members of bacterial flora on the oral mucosa^35^. Such abnormal cytoskeletal arrangement may also be considered to be due to keratinocyte damage or activation^22^, though the stimulating factors in cases of oral lichen planus are unknown. Previous electron microscopy studies have disclosed increased thickening and granularity of tonofibrils in oral lichen planus^27–30^. In findings presented by Griffin et al., clumped tonofilaments were shown to be engulfed by macrophages and some of the phagocytosed epithelial material could be released into the lamina propria^27^, likely because they are not digestible. This indigestible nature of K17+ filamentous conglomerates would enable Civatte bodies to have extended survival and be easily recognized by pathologists. However, in the present investigation, we did not find any Civatte bodies phagocytosed by macrophages, which were instead shown to frequently engulf apoptotic bodies. Although apoptotic oral SCC cells are often engulfed by neighboring SCC cells^36^, Civatte bodies are not able to phagocytose apoptotic cells because they are just denucleated cell ghosts/debris.

It is important to note that Civatte bodies do not represent apoptotic figures because they lack TUNEL signals, which must be a natural condition because of their lack of nuclei. Although Cheng et al. described Civatte bodies as representing keratinocyte apoptosis in a position paper of the American Academy of Oral and Maxillofacial Pathology for oral lichen planus diagnosis^11^, we found no evidence in that article that they are derived from apoptotic cells. In a further search of relevant literature, there was no documentation showing apoptotic evidence for Civatte bodies, while the present results are the first to demonstrate that Civatte bodies are not apoptotic products. Our findings indicating absence of TUNEL signals within Civatte bodies and their K17 positivity are identical to the first electron microscopic studies conducted by Ebner and Mehregan, which demonstrated that Civatte bodies contained no nuclear components, but were exclusively composed of 8- to 10-nm (intermediate keratin) filaments^13^. Based on the present results, it is possible to consider that Civatte bodies are not in an apoptotic process but rather generated as a part of an unknown cell death process different from conventional apoptosis or necrosis, as an irregular process of single cell keratinization expressing K17 during which nuclei are deleted. It is difficult to explain the caspase 3 positivity in Civatte bodies shown in the present study, but it is unlikely that is simply due to its retention in filamentous aggregates, even after the autophagocytotic processes of macrophages.

Hemoglobin labelling in Civatte bodies suggests a possible histopathogenesis triggered by collapse of blood capillary loops in the lamina propria. Based on their results of Ig+ Civatte bodies that had absorbed Ig, Biermann and Rauterberg suggested that Civatte bodies can naturally absorb soluble molecules that are retained in the local milieu^16^. In addition to Ig, hemoglobin caused by hemolysis of extravasated erythrocytes from collapsed vessels must spontaneously enter Civatte bodies, which have lost their cell membranes^13^, and be condensed within them. In other words, Civatte bodies are soaked in soluble blood contents. We speculate that vascular collapse is due to lymphocytic infiltration, though the detailed cellular mechanism of lichen planus remains largely unknown. Nevertheless, as we previously showed in an examination of oral SCC cell lines, oxidation by extravasated erythrocytes or hemoglobin induces keratinization of carcinoma cells^23^. Thus, exposure of prickle cells to hemorrhage may be the general background for their K17 expression, eventually leading to K17+ Civatte bodies. We also consider that hemorrhage and hemosiderosis following blood capillary collapse are important histopathological events in oral lichen planus that have not yet attracted attention as possible histopathogenesis factors.

## Conclusions

Civatte bodies of oral lichen planus are clearly demonstrable by their strong K17 immunopositivity, and they are not apoptotic products but rather the result of an unknown type of single cell keratinization process that includes denucleation and loss of cell membranes from K17+ epithelial cells. This unusual course of keratinization is likely attained in a process different from normal, starting from K13+ prickle cells of the oral mucosa. Squamous epithelial cells affected by oral lichen planus contain irregularly arranged K17+ filaments and have lost K13+ filaments. However, the switch from K13 to K17 in oral lichen planus is only halfway completed, whereas oral malignancy shows full switching.

## Data Availability

The experimental data analyzed during the current study are available from the corresponding author on reasonable request.
